# Impact of psoriasis disease activity and other risk factors on serum urate levels in patients with psoriasis and psoriatic arthritis—a *post-hoc* analysis of pooled data from three phase 3 trials with secukinumab

**DOI:** 10.1093/rap/rkab009

**Published:** 2021-02-18

**Authors:** Mats Dehlin, Andreas E R Fasth, Maximilian Reinhardt, Lennart T H Jacobsson

**Affiliations:** 1 Department of Rheumatology and Inflammation Research, Institute of Medicine, Sahlgrenska Academy, University of Gothenburg, Gothenburg, Sweden; 2 Medical Affairs, Novartis Sverige AB, Kista, Sweden; 3 Novartis Pharma AG, Basel, Switzerland

**Keywords:** psoriasis, urate, hyperuricaemia, risk factors, gout

## Abstract

**Objectives:**

Our aims were to determine if the Psoriasis Area Severity Index (PASI) score and serum urate (SU) levels were associated at baseline and whether the change in PASI score during 12 weeks of treatment resulted in a significant change in SU, adjusted for relevant confounders.

**Methods:**

Data from patients with psoriasis/PsA (*n* = 1042/204) in three phase 3 randomized control trials treated with secukinumab (dose 300 mg, *n* = 628) or placebo (*n* = 414) were pooled. At baseline, values for SU, PASI and the following covariates were assessed: age, sex, BMI, estimated glomerular filtration rate, and medication with diuretics. To assess the changes in PASI (ΔPASI) and SU (Δurate), the differences (week 12 minus baseline) in patients receiving the active drug were used. Multivariable linear regression, adjusting for covariates, was used to assess the association between PASI and SU at baseline with all patients pooled and to assess the association between Δurate and ΔPASI over 12 weeks of treatment with secukinumab.

**Results:**

The degree of skin involvement of psoriasis showed a statistically significant, albeit modest, association with SU (*R*^2^ = 0.014, *P* < 0.0001 univariately), whereas known risk factors for hyperuricaemia had a much larger impact cross-sectionally at baseline (*R*^2^ = 0.33, *P* < 0.0001). Furthermore, a substantial improvement in PASI score resulted in only a modest decrease of SU over 12 weeks of treatment with secukinumab (*R*^2^ = 0.014, *P* < 0.0001 univariately).

**Conclusions:**

There is a statistically significant, albeit modest, association with both extent and change in PASI score and SU in patients with psoriasis, compatible with a potential pathophysiological relationship between urate and psoriasis.

**Trial Registration:**

ERASURE: clinicaltrials.gov, https://clinicaltrials.gov, NCT01365455; FIXTURE: clinicaltrials.gov, https://clinicaltrials.gov, NCT01358578; SCULPTURE: clinicaltrials.gov, https://clinicaltrials.gov, NCT01406938

## Introduction

Key messages

- Known associations between urate and its known risk factors are valid in patients with psoriasis.
- Disease activity in psoriasis is associated with urate blood levels.
- There is a potential pathophysiological relationship between urate and psoriasis.

Elevated serum urate level, hyperuricaemia, is associated with increased risk for conditions severely impacting peoples’ lives, including chronic kidney disease, cardiovascular disease and gout [1]. Urate blood levels are dependent on secretion by urate transporters in the kidneys and the gut. Over recent years, it has been shown that variation in the genes coding for these transporters are the main determinants for urate blood levels [2]. Decrease in kidney function and use of alcohol and diuretics have a negative impact on renal secretion of urate, resulting in hyperuricaemia [3, 4]. Blood levels of urate are also affected by a diet rich in purines (beer, seafood and organ meats) [5] and by conditions with increased cell turnover (haematological malignancies) through extracellular release of purine-rich components, such as DNA [6]. Additionally, age, male sex, hypertension and obesity are the most well-known factors associated with hyperuricaemia, although the underlying mechanisms are not fully understood [7, 8].

Psoriasis is an immune-mediated skin disease. Apart from genetic susceptibility, known risk factors for psoriasis are mechanical stress, drugs, smoking, air pollutants and exposure to ultraviolet radiation [9]. Hyperuricaemia has been reported to be associated with psoriasis [10], and both conditions are associated with chronic kidney disease, hypertension, obesity and metabolic syndrome; therefore, they might be confounders for an association [11, 12]. Furthermore, obesity is strongly associated with the onset and exacerbation of psoriasis [9] in addition to increased levels of urate [8]. There is some previous support that gout, with the hallmark hyperuricaemia, is associated with a significantly increased risk of development of psoriasis [13] and that patients with psoriasis exhibit an elevated risk for development of gout [14].

Hyperuricaemia and psoriasis share many risk factors for cardiovascular disease, such as hypertension and obesity, but also inflammation. Urate may give rise to inflammation through activation of the NLRP3 inflammasome, as in gout, followed by production of the pro-inflammatory cytokine IL-1β [15], whereas psoriasis is characterized by other inflammatory cytokines, such as TNF-α, IL-23 and IL-17 [16].

The increased cell turnover in psoriatic skin lesions has been proposed as a potential mechanism for the elevated serum urate levels associated with psoriasis, but results are conflicting [10]. Thus, it is unclear whether or to what degree an association exists.

In this study, we investigated the hypothesis that psoriasis disease severity, as reflected by the level and change in the Psoriasis Area Severity Index (PASI) score, is associated with serum urate levels in the context of data collected in three large, pivotal randomized control trials of secukinumab, an IL-17A inhibitor, for psoriasis. Our aims were to determine whether the PASI score and serum urate levels were associated cross-sectionally at baseline, and whether a change in PASI score during 12-week treatment was associated with a significant change in serum urate, adjusted for relevant confounders.

## Materials and methods

### Study population

A total of 1042 patients with psoriasis with or without concomitant PsA [*n* = 204/1042 (19.6%)] in three pivotal randomized control trials (ERASURE, FIXTURE and SCULPTURE) [17, 18] treated with secukinumab (300 mg/month, *n* = 628) or placebo (*n* = 414) were pooled and included. Patients treated with 150 mg secukinumab per month were not included in the present study, because the dose approved for the treatment of psoriasis is 300 mg, and this dose shows greater efficacy on the PASI score. For more information of the three study populations, see Supplementary Table S1, available at *Rheumatology Advances in Practice* online.

### Study conduct

The study protocols for the three randomized control trials were approved by the institutional review board or ethics committee at each participating site, and the studies were conducted in accordance with the ethical principles of the Declaration of Helsinki. The US sites maintained compliance with Health Insurance Portability and Accountability Act regulations. Eligible patients provided written informed consent.

### Assessment of psoriasis, urate and confounders

The eligibility criteria were similar in the three studies. All participants were aged ≥18 years, with moderate-to-severe, poorly controlled plaque psoriasis that had been diagnosed ≥6 months before randomization. At inclusion, patients had a PASI score of ≥12 [17, 18].

In the placebo group, values for serum urate and PASI scores were assessed at baseline and week 12, whereas in the secukinumab group it was also recorded at weeks 16, 24, 32, 40, 48 and 52. At baseline, the following covariates known to affect urate levels were assessed: age, sex, BMI, renal function defined as estimated glomerular filtration rate (eGFR) and medication with diuretics (%) [17, 18].

The PASI combines measures of redness, thickness and scaliness of lesions (each on a scale from zero to four: none, slight, mild, moderate or severe) with the amount of body surface area affected (on a scale from one to six). The lesional characteristics and the body surface area involved are measured separately for the head, upper extremities, trunk and lower extremities. The resulting scale ranges from 0 to 72. A PASI score >12 is frequently used as a criterion for severe disease [19].

### Statistics

Descriptive statistics are presented as the number (percentage) or mean (s.d.). For baseline association analysis, uni- and multivariable linear regression, adjusting for identified covariates, were used to assess the relationship between PASI and urate at baseline, with all patients (placebo and secukinumab) pooled.

For follow-up association analysis of changes in PASI (ΔPASI) and urate (Δurate), the differences (week 12 minus baseline) in patients receiving active treatment (i.e. secukinumab) were used. Uni- and multivariable linear regression, adjusting for identified covariates, was used to assess the associations between Δurate and ΔPASI over the 12-week intervention.

For baseline and follow-up association analysis, all independent continuous variables (age, BMI, eGFR and PASI) were standardized using the s.d. for the whole population at baseline (*n* = 1042). This was to enhance comparability of the effects from the different covariates on urate. Thus, unit-wise increments in parameter estimates for each continuous covariate represented the change in urate per 1 s.d. at baseline. The dependent variable, serum urate, was not standardized. The statistical program used was SAS v.9.4 (SAS Institute Inc., Cary, NC, USA).

## Results

The baseline characteristics of the total group (secukinumab and placebo combined) were similar to both the secukinumab group and the placebo group; 70–71% males and 6–7% diuretic users (Table 1). The groups were also similar with regard to the mean (s.d.) age 45 (13) years, BMI 29 (7) kg/m^2^, eGFR 96–97 (17) ml/min/1.73 m^2^, PASI 23.2–23.7 (10) and urate 359–366 (93–96) µmol/l respectively, (Table 1).

**Table 1:** Baseline characteristics of the total study population and the subgroups treated with placebo and secukinumab (300 mg)

Variable	Baseline placebo, *n* = 414	Baseline secukinumab, *n* = 628	Baseline secukinumab + placebo, *n* = 1042
Age, years, mean (s.d.)	45.3 (13)	45.5 (13)	45.4 (13)
Sex, male, *n* (%)	285 (69)	447 (71)	732 (70)
BMI, kg/m^2^, mean (s.d.)	29.3 (7)	29.1 (7)	29.2 (7)
eGFR, ml/min/1.73 m^2^, mean (s.d.)	97 (18)	96 (17)	96 (18)
Diuretics user, yes, *n* (%)	25 (6)	42 (7)	67 (6)
PASI, mean (s.d.)	22.5 (10)	23.7 (10)	23.2 (10)
Urate, µmol/l, mean (s.d.)	359 (90)	366 (96)	363 (93)

Values are given as the mean (s.d.). eGFR: estimated glomerular filtration rate; PASI: Psoriasis Area Severity Index.

**Fig. 1:**
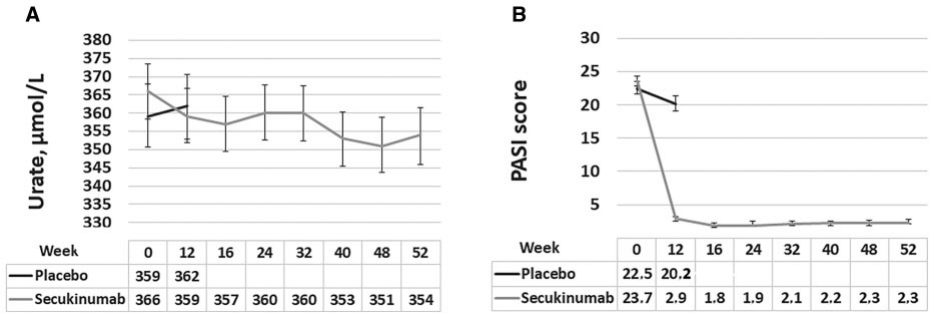
Urate and Psoriasis Area Severity Index score at weeks 0 and 12 for placebo and to week 52 for secukinumab-treated patients (**A**) Serum urate at weeks 0 and 12 for the placebo group and at intervals between baseline and week 52 for the secukinumab-treated group with 95% CIs. Means of urate (in micromoles per litre) are given in the table below. (**B**) PASI score at weeks 0 and 12 for the placebo group and at intervals between baseline and week 52 for the secukinumab-treated group with 95% CIs. Means of PASI score are given in the table below. PASI: Psoriasis Area Severity Index.

From baseline to week 12 in patients treated with secukinumab, the mean (s.d.) PASI decreased from 23.7 (10) to 2.9 (5) and mean urate (s.d.) from 366 (96) to 359 (94) µmol/l (Fig. 1). These improvements were slightly amplified after 52 weeks of secukinumab treatment, with mean (s.d.) PASI 2.3 (4.5) and urate mean (s.d.) 353.7 (90.1) µmol/l (Fig. 1). In the placebo group, from baseline to week 12, the mean (s.d.) PASI decreased from 22.5 (9.9) to 20.2 (12.2), and mean urate (s.d.) increased from 359.4 (89.9) to 361.8 (92.8) µmol/l (Fig. 1).

**Fig. 2:**
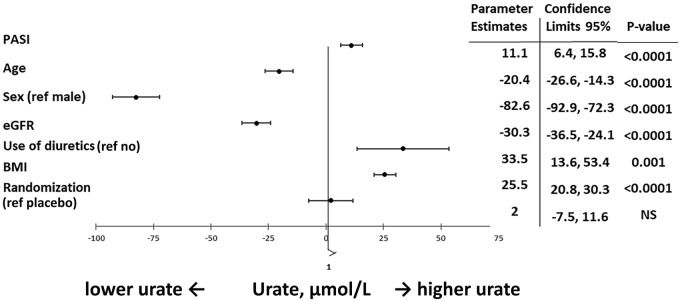
Multivariable linear regression models, with urate at baseline as the dependent variable, in secukinumab and placebo groups *n* = 1042. All continuous variables were standardized, with the exception of the dependent variable, urate. eGFR: estimated glomerular filtration rate; NS, not significant.

At baseline, covariates explained 33% of the variance in urate (*R*^2^ = 0.33, *P* < 0.0001), but univariately PASI alone explained only 1.4% of the variance of urate (*R*^2^ = 0.014, *P* < 0.0001; Table 2). All covariates, including the PASI, were significantly associated with urate in both univariate and multivariate analyses. A PASI higher than 1 s.d. (10 units) was associated with 11.2 µmol/l higher value of urate in the fully adjusted model (*P* < 0.0001; Fig. 2). However, factors known to affect urate had an even larger impact. A decrease in kidney function of 1 s.d. eGFR (18 ml/min/1.73 m^2^) and a higher value of 1 s.d. BMI (7 kg/m^2^) were associated with 30 µmol/l (*P<* 0.0001) and 25.5 µmol/l (*P* < 0.0001) higher value of urate in the fully adjusted model, respectively (Fig. 2). Furthermore, male sex and the use of diuretics, both well-known risk factors for hyperuricaemia, had even larger effects on urate in the fully adjusted model: 82.6 µmol/l (*P* < 0.0001) and 33.5 µmol/l (*P* = 0.001), respectively (Fig. 2).

**Table 2:** Univariate linear regression models, with urate at baseline as the dependent variable, in the total group (secukinumab and placebo), *n* = 1042

Variable	Point estimate	95% CL	*P*-value	*R* ^2^
Per 1 s.d. PASI, (s.d. = 10)	11.2	5.6, 16.8	0.0001	0.014
Per 1 s.d. Age, years (s.d. = 13)	2.0	−3.7, 7.7	NS	0.000
Sex (reference male)	−82.1	−93.5, −70.8	<0.0001	0.162
Per 1 s.d. eGFR, (ml/min/1.73 m^2^), (s.d. = 18)	−20.0	−25.6, −14.5	<0.0001	0.046
Diuretics (reference no)	52.5	29.5, 75.4	<0.0001	0.019
Per 1 s.d. BMI (kg/m^2^) (s.d. = 7)	25.4	19.9, 30.8	<0.0001	0.074
Randomization (reference placebo)	6.5	−5.1, 18.1	NS	0.001

All continuous variables were standardized, with the exception of the dependent variable urate, and the parameter estimate represents the effect on urate of change per 1 s.d. CL: confidence limits; eGFR: estimated glomerular filtration rate; NS: not significant; PASI: Psoriasis Area Severity Index.

During 12 weeks of anti-psoriatic treatment with secukinumab, covariates overall explained little of the variation in Δurate (*R*^2^ = 0.16), although there was a significant association with ΔPASI in the fully adjusted model (*P* < 0.0001; Table 3). In the unadjusted model, change in PASI explained little of the variance in change of urate (*R*^2^ = 0.04, *P* < 0.0001). A decrease in ΔPASI value of 1 s.d. (13) was associated with a decrease of Δurate with 15.5 µmol/l (*P* < 0.0001) in the univariate model and 12.0 µmol/l (*P* < 0.0001) in the fully adjusted model (Table 3). Thus, a substantial reduction in PASI over 12 weeks was associated with a small but significant reduction of urate.

**Table 3:** Uni- and multivariable linear regression models, with Δurate (week 12 minus week 0) as the dependent variable, in patients treated with secukinumab, *n* = 628

ΔPASI	Parameter estimate	95% CL	*P*-value	*R* ^2^
ΔPASI (week 12 minus baseline)^1^	15.5	9.8, 21.1	<0.0001	0.04
ΔPASI (week 12 minus baseline)^2^	15.1	9.5, 20.8	<0.0001	0.05
ΔPASI (week 12 minus baseline)^3^	16.0	10.3, 21.7	<0.0001	0.06
ΔPASI (week 12 minus baseline)^4^	12.0	6.5, 17.5	<0.0001	0.16

All continuous variables were standardized, with the exception of the dependent variable, urate. Thus, unit-wise increments in parameter estimates for 1 s.d. of ΔPASI (13) represent the change in Δurate (in micromoles per litre). The superscripts 1–4 indicate the four different models of adjustment: model 1, univariate linear regression with 1 s.d. ΔPASI (week 12 minus week 0); model 2, multivariable linear regression with 1 s.d. ΔPASI (week 12 minus week 0) adjusting for age and sex; model 3, multivariable linear regression with 1 s.d. ΔPASI (week 12 minus week 0) adjusting for age, sex, eGFR, diuretic use and BMI at baseline; and model 4, multivariable linear regression with 1 s.d. ΔPASI (week 12 minus week 0) adjusting for age, sex, eGFR, diuretic use, BMI and urate at baseline. CL: confidence limits; eGFR: estimated glomerular filtration rate; PASI: Psoriasis Area Severity Index.

## Discussion

In the present study, we showed that both degree and change in skin involvement in psoriasis had a statistically significant, but modest, association with urate, whereas known risk factors for hyperuricaemia had a much larger impact cross-sectionally at baseline. Furthermore, a substantial improvement in PASI score resulted in only a modest decrease of serum urate levels over 12 weeks of treatment with secukinumab.

Previously reported associations between psoriasis and urate are conflicting. A meta-analysis by Li *et al.* [10] identified 14 observational studies on urate and psoriasis. Li *et al.* [10] reported significantly increased levels of urate in psoriasis in studies performed in western Europe but not in East Asia, India or the Middle East, suggesting differences related to ethnicity or region. Our study was based on the 1042 participants of three secukinumab trials (ERASURE, FIXTURE and SCULPTURE) [17, 18], who were recruited at 452 sites in six continents and thus include a mixture of different ethnicities. It was beyond the scope of the present analyses to investigate whether effects varied across ethnicities; this will need to be addressed in future studies.

Hyperuricaemia and psoriasis have many risk factors in common, which might confound the association. BMI is not only higher in patients with psoriasis [20] and positively correlated with the severity of the psoriatic skin manifestations [21], but also exhibits a high positive correlation with urate levels [8]. Psoriasis is associated with an increased risk of chronic kidney disease and end-stage renal disease [22], both of which lead to decreased secretion of urate and development of hyperuricaemia. Furthermore, cardiovascular disease is also more common in psoriasis [23], and the increased occurrence of chronic kidney disease and cardiovascular disease implies an increased use of diuretics, which is a well-known cause of hyperuricaemia. However, when adjusting our analyses for these potential confounders, we still found a small but significant association between PASI and urate levels, supporting a potential pathophysiological relationship, possibly explained by increased cell turnover in psoriatic skin lesions. Could the relationship be reversed? It is beyond the scope of this study to provide an answer, but urate has historically been suggested to be a causative agent for psoriasis [24], possibly by activation of plasmacytoid dendritic cells and keratinocytes [25].

The most well-known consequence of hyperuricaemia is gout, which varies greatly in prevalence over the continents of the world. This has historically been attributed to differences in culture and lifestyle, but in recent years variations in the genes regulating secretion of urate by the kidneys and the gut have been acknowledged as a major explanatory factor for the observed variation [2]. Variations in genes regulating immunity and skin barrier processes have been shown to be risk factors for psoriasis [26]. The occurrence of psoriasis also varies with geographical region; for example, it occurs more frequently in countries more distant from the equator [27]. However, these variations seen worldwide for hyperuricaemia/gout and psoriasis do not overlap [27, 28]. Consequently, it is less likely that there are other unknown shared aetiological factors between psoriasis and hyperuricaemia/gout that were not included in the present study.

Another interesting aspect of uric acid and hyperuricaemia is the role of urate as an activator of the NLRP3 inflammasome, and thereby of IL-1β production. In addition, there are also inflammasome-independent pathways through which urate crystals can trigger IL-1β production. The resulting bioactive IL-1β stimulates the inflammation of gout and might contribute to the development of other co-morbidities [29]. Hyperuricaemia is associated with a number of co-morbidities, such as renal disease, type 2 diabetes, metabolic syndrome, atherosclerosis or other cardiovascular diseases, such as hypertension. For chronic kidney disease, urate might be both a cause and a consequence. For other co-morbidities, such as cardiovascular disease, associations with serum urate are consistent, but a causal relationship is less clear. The exact mechanism showing how uric acid mediates these co-morbidities is incompletely understood; however, it is becoming more evident that the sterile inflammation caused by hyperuricaemia via triggering of IL-1β production is a key process [29]. Atherosclerosis, type 2 diabetes or metabolic syndrome are today understood as sterile inflammatory diseases, with inflammatory processes in the vascular endothelium or in adipose tissue being cornerstones of their pathogenesis [29]. Therefore, uric acid-triggered IL-1β-dependent inflammation might be a key link between hyperuricaemia and its co-morbidities [15]. Interestingly, many of these cardiometabolic co-morbidities, which are increasingly understood as inflammatory diseases, are also highly prevalent in the psoriasis patient population, as mentioned in the Introduction. Further work is needed to determine whether hyperuricaemia plays a role in the systemic inflammation and cardiometabolic co-morbidity seen in psoriasis patients.

There are several strengths to the present study. First is the high validity of the psoriasis diagnosis in the included study patients. Second is the large sample size with systematic information on PASI, urate and relevant confounders. Third is the longitudinal data, which allowed us to analyse associations between the change in urate and PASI score. A possible limitation to the study is the lack of information on possible urate-lowering treatment in the study patients, although the age span of the study is well below the peak age for the incidence of gout.

In conclusion, the present study showed that the known associations between urate and many of its known risk factors are valid in psoriasis. In addition, we found a statistically significant, but modest, association between psoriasis disease activity and change in psoriasis disease activity and urate blood levels, compatible with a potential pathophysiological relationship between urate and psoriasis. Future research should focus on the role of hyperuricaemia on both the risk and extent of psoriasis and its role in systemic inflammation and cardiometabolic co-morbidity in psoriasis.

*Funding*: Novartis was the funding source for this study. The statistical analysis was performed by Novartis PLS Statistics Team, Novartis Ireland Limited, Dublin, Ireland. All researchers assigned as authors and not affiliated to Novartis state their complete independence from the funders, with regard to this study.

*Disclosure statement*: M.R. is an employee of Novartis. A.E.R.F. was an employee of Novartis during the conduct and analysis of the study. The other authors have declared no conflicts of interest.

## Data availability statement

The data underlying this article were provided by Novartis by permission. Data will be shared on request to the corresponding author with permission of Novartis.

## Supplementary data

Supplementary data are available at *Rheumatology Advances in Practice* online.

## Supplementary Material

rkab009_Supplementary_Data
