# Supplementary material for: Impact of psoriasis disease activity and other risk factors on serum urate levels in patients with psoriasis and psoriatic arthritis—a post-hoc analysis of pooled data from three phase 3 trials with secukinumab
Source: Rheumatol Adv Pract. 2021 Feb 18;5(1):rkab009. doi: 10.1093/rap/rkab009 (PMC7962727; doi:10.1093/rap/rkab009)
Supplement: rkab009_Supplementary_Data [file rkab009_supplementary_data.docx]

|  | ERASURE | | FIXTURE | | SCULPTURE |
| --- | --- | --- | --- | --- | --- |
|  | Secukinumab 300 mg, n=245 | Placebo, n=248 | Secukinumab 300 mg, n=327 | Placebo, n=326 | Secukinumab 300 mg, n=484 |
| Age, years, mean (SD) | 44.9 (13.5) | 45.4 12.6) | 44.5 (13.2) | 44.1 (12.6) | 46.7 (12.8) |
| Gender, male, n (%) | 169 (69.0) | 172 (69.4) | 224 (68.5) | 237 (72.7) | 333 (68.8) |
| Race, n (%) |  |  |  |  |  |
| Caucasian/White | 171 (69.8) | 176 (71) | 224 (68.5) | 218 (66.9) | 343 (70.9) |
| Asian | 52 (21.2) | 46 (18.5) | 73 (22.3) | 72 (22.1) | 123 (25.4) |
| Other/Unknown | 22 (9.0) | 26 (10.5) | 30 (9.2) | 36 (11.0) | 18 (3.7) |
| Weight, kg (SD) | 88.8 (24) | 89.7 (25) | 83 (21.6) | 82 (20.4) | 85.1 (23.2) |
| BMI, (SD) | 30.3 (7.2) | 30.3 (7.8) | 28.4 (6.4) | 27.9 (6.1) | 29.0 (6.88) |
| Time since psoriasis diagnosis, years, mean (SD) | 17.4 (11.1) | 17.3(12.4) | 15.8 (12.3) | 16.6 (11.6) | 17.4 (12.88) |
| PASI score, mean (SD) | 22.5 (9.2) | 21.4 (9.1) | 23.9 (9.9) | 24.1 (10.5) | 23.3 (9.6) |

**Supplementary Table S1. Baseline characteristics for the participants in the three secukinumab RCTs ERASURE, FIXTURE and SCULPTURE receiving secukinumab 300 mg or placebo.**

*SD*, standard deviation; *PASI*, psoriatic arthritis severity index.
